# The Escherichia coli F plasmidome: an Australian perspective

**DOI:** 10.1099/mgen.0.001669

**Published:** 2026-04-24

**Authors:** Max L. Cummins, Anne E. Watt, Celeste M. Donato, Amy V. Jennison, Torsten Seemann, Erica Donner, Cameron J. Reid, Danielle J. Ingle, Benjamin P. Howden, Steven P. Djordjevic, on behalf of the Australian Pathogen Genomics One Health Working Group

**Affiliations:** 1 Australian Institute for Microbiology and Infection, University of Technology Sydney, Ultimo, Australia; 2 The Australian Centre for Genomic Epidemiological Microbiology, University of Technology Sydney, Ultimo, Australia; 3 Microbiological Diagnostic Unit, Public Health Laboratory, Peter Doherty Institute for Infection & Immunity, University of Melbourne, Melbourne, Victoria, Australia; 4 Department of Microbiology and Immunology, Peter Doherty Institute for Infection & Immunity,, University of Melbourne, Melbourne, Victoria, Australia; 5 Centre for Pathogen Genomics, Department of Microbiology and Immunology, University of Melbourne, Victoria, Australia; 6 Public and Environmental Health, Pathology Queensland, Queensland Health, Brisbane, Australia; 7 Future Industries Institute, Adelaide University, Mawson Lakes, SA 5095, Australia; 8 Cooperative Research Centre for Solving Antimicrobial Resistance in Agribusiness, Food, and Environments, Mawson Lakes, SA 5095, Australia; 9 Microba Life Sciences Ltd, Brisbane, Australia

**Keywords:** ColV, *Escherichia coli*, F plasmids, One Health, plasmids, pUTI89

## Abstract

ColV/ColBM and ColIa/*senB* F virulence plasmids feature prominently in *Escherichia coli* associated with urinary tract and bloodstream infections globally. Australian-sourced *E. coli* that carry these plasmids were examined among 5,471 isolates (3,316 sequenced by the APG and AusGEM programmes; 2,155 from public databases) spanning years 1986–2020 from humans (*n*=2,996/5,471; 54.8%), wild animals (*n*=870/5,471; 15.9%), livestock (*n*=649/5,471; 11.9%), companion animals (*n*=375/5,471; 6.9%), environmental sources (*n*=292/5,471; 5.3%) and food (*n*=289/5,471; 5.3%). Putative plasmid reconstruction, assisted by a plasmid database comprising 23,700 complete plasmid sequences, identified 22,534 putative plasmids of which 21,814 (96.8%) represented 547 known plasmid clusters. *E. coli* harbouring ColV-associated putative plasmids, particularly plasmid cluster AA176 [F replicon sequence type (RST): F18:A-:B1 (repFII-18:repFIA-null:repFIB-1)] was identified among phylogenetically diverse strains from humans, livestock, particularly poultry, and food. Closely related isolates, defined as ≤10 core-genome multilocus sequence type allelic distance, that carried either ColV or ColIa/*senB*-associated putative (F) plasmids were identified across multiple sources and diverse phylogenetic backgrounds. ColIa/*senB*-associated putative (F) plasmid clusters AA337 (RST: F29:A-:B10) and AA171 (RST: F2:A1:B20) were associated with phylogenetically closely related isolates from humans, wild animals and companion animals, but their absence in *E. coli* sourced from food and livestock was notable. *E. coli* carrying ColV and ColIa/*senB* plasmids frequently exhibit genotypic multidrug resistance, many with critically important antimicrobial resistance genes, highlighting their role in the evolution of clinically problematic lineages. Our study has important epidemiological considerations for understanding the spread of extraintestinal pathogenic and hybrid *E. coli* lineages across the One Health spectrum.

**Impact Statement**

This study provides the most comprehensive genomic analysis to date of virulence-associated *Escherichia coli* plasmids circulating in Australian *E. coli*, collected from diverse ecological niches over nearly four decades. Combining detailed genomic analysis of 5,471 genomes and 23,000 reference plasmids, and through leveraging recent advances in plasmid clustering approaches, we demonstrate that ColV and ColIa/*senB* plasmids bridge humans, animals, food, wildlife and the environment and act as key vehicles for virulence and antimicrobial resistance across the One Health interface. The identification of key plasmid clusters critical in both human and animal extraintestinal infections, and their association with particular ecological niches, has significant public health implications, as these dynamics shape the evolution and global dissemination of pandemic and emergent extraintestinal pathogenic *E. coli* lineages.

## Data Summary

Genomic data utilized in this study is available at the NCBI Sequence Read Archive (https://www.ncbi.nlm.nih.gov/sra), with SRA accession numbers for individual genomes provided in Supplementary Text 1. Scripts used to process and visualize data are available on Github https://www.github.com/maxlcummins/APG-OHEC-Retro-M2.

## Introduction

*Escherichia coli* is a frequently isolated bacterial pathogen, increasingly associated with morbidity and mortality caused by multiple drug-resistant (MDR) strains [1], and presents as one of the most challenging disease-causing Gram-negative bacterial species to impact human and animal health. Two primary pathotypes of this species exist: intestinal pathogenic *E. coli*, which cause diarrhoeal and other gastrointestinal diseases, and extraintestinal pathogenic *E. coli* (ExPEC), which are primarily associated with urinary tract infection (UTI) and sepsis. *E. coli* is renowned for its ability to assemble a remarkable repertoire of laterally acquired antimicrobial resistance genes (ARGs) localized within complex resistance regions [2] as well as an extraordinary capacity to acquire and retain virulence gene cargo. The emergence of hybrid pathovar variants [3,7] that combine key intestinal and extraintestinal virulence genes [8] complicates *E. coli* disease diagnosis and treatment. In this regard, F plasmids play an important role where they capture novel combinations of virulence gene cargo, often in association with ARGs [6,9].

F plasmids are narrow host range, large (>100 kb), independently replicating, conjugative genetic elements originally defined by the fertility (F) factor of *E. coli*. They are characterized by an IncF replicon, complex multi-replicon architectures and a conserved conjugative transfer system [10]. Their ability to carry significant virulence and ARG cargo is pivotal to the success of ExPEC [10] including pandemic lineages ST131 [11], ST1193 [12], ST410 [13,15], ST95 [9] and others [16,17]. Significant in this regard are epidemic F resistance plasmids with divergent replicon types [18,20] that have acquired multiple resistance genes and disseminate them across the *Enterobacteriaceae*, particularly multi-replicon FII plasmids and their importance in the global dissemination of *bla*_CTX-M_ genes [21,22], which encode resistance to critically important third-generation cephalosporins. F plasmids influence the ecology and evolution of MDR and pathogenic *E. coli* lineages [10,16] with their capacity to mediate the transfer of large chromosomal fragments [23] considered seminal in the evolution of numerous pandemic enterobacterial clones including ExPEC pandemic lineages ST131 [11], ST410 [24] and ST1193 [25,26].

*bla*_CTX-M_ genes, particularly *bla*_CTX-M-15_, are strongly associated with F plasmids to the extent that these plasmids are often referred to as epidemic resistance plasmids [19]. These elements often house complex resistance regions and are notable for their ability to disseminate rapidly among members of the *Enterobacteriaceae*. F plasmids carrying *bla*_CTX-M-15_ increased in frequency during the mid-2000s, and this extended-spectrum beta-lactamase encoding gene has become dominant in the global F plasmid landscape [19]. F plasmids carrying *bla*_CTX-M-15_ are frequently encountered in ST131 clade C2 [27], ST1193 [12] and other pandemic ExPEC sequence types (STs) such as ST410 [24] and ST95 [9]. F plasmids are also important disseminators of ARGs in *E. coli* that colonize the healthy human microbiome [28] and food-producing animals [29].

Recently, we performed a clustering analysis of predicted F plasmids found in 4,711 bloodstream infection (BSI) *E. coli* draft genomes derived from public database repositories [30]. Based on a model of complete F plasmid sequences, our analyses identified five F plasmid clusters of which two were dominant in the collection, representing almost 50% of the clustered sequences [30]. Of the two dominant clusters, one was represented by F plasmids (RSTs F24:A-:B1, F2:A-:B1, F18:A-:B1 and F36:A6:B1) carrying ColV-like virulence markers including those involved in iron acquisition (salmochelin; aerobactin and *ets* and *sit* operons) and serum survival (*iss*), TSH (toxin secretion operon) and haemolysin (HlyA) [31,32]. The other dominant cluster was represented by F plasmids carrying *cjrABC-senB* [33], ‘island 1’ which contains a suite of iron binding and transport genes, and a putative hemin receptor, all of which are markers for pUTI89 (F29:A-:B10) and related (F1:A1:B20, F-:A1:B10, F2:A1:B-; F51:A-:B10; F31:A4:B1, F36:A4:B1 and F51:A-:B10) F plasmids [12,30, 34,36]. These two F plasmid families are linked with specific ExPEC sublineages found in many of the top 20 ExPEC STs responsible for UTI and BSI [37] including ST131, ST1193, ST69, ST38, ST95, ST405, ST648, ST127, ST88, ST58 and ST73 [12,17, 30, 38,43]. *E. coli* genomes with F plasmids carrying *senB* were abundant in our analysis of BSI isolates, present in 27.4% of all sequences (*n*=1,290/4,711), while *E. coli* carrying ColV F plasmids were identified in 19.6% (*n*=924/4,711) of the collection [30] underscoring the importance of these F plasmid lineages in extraintestinal disease.

A recent longitudinal epidemiological study spanning 16 years and involving 3,245 BSI *E. coli* isolates in Norway [44], underpinned by long-read sequencing of 2,045/3,245 isolates (62.9% of the NORM collection) representing 216 distinct STs across the ExPEC phylogeny, identified widespread carriage of F plasmids with ColV and *senB* markers, highlighting their important role in the evolution of pandemic ExPEC lineages and in the clonal expansion of AMR [38]. Significant in this regard is the acquisition of ARGs often flanked by copies of IS*26* in both these virulence plasmid lineages [12,38]. Collectively, plasmid studies of *E. coli* recovered from patients with BSI [30,38, 45] and UTIs [17] from different European countries and in Australia have firmly established the importance of F plasmids carrying ColV and *senB* virulence gene cargo.

Recently, we analysed a large national, multi-sectoral genomic dataset comprising 5,471 *E. coli* genomes to identify cross-source genomic clusters with the overarching aim to better define relevant measures of genomic relatedness in a One Health context in Australia [46]. Here, we have interrogated this retrospective national genomic dataset to investigate carriage of F plasmids virulence gene cargo from a One Health perspective.

## Methods

### Genome collation

We collated a large One Health *E. coli* genome collection as previously described [46]. Note that due to the association of many of the genomes under analysis with public health laboratories and veterinary collections associated with food animal outbreaks, the collection is intrinsically biassed towards clusters of closely related genomes. All genomes under analysis were generated using short-read sequencing platforms; metadata, genotypic data, bioproject and accession numbers for genomes under analysis are available in Table S1 (available in the online Supplementary Material) (https://github.com/maxlcummins/APG-OHEC-Retro-M2).

### Companion scripts

Genomes were analysed using a Snakemake [47] pipeline available at https://www.github.com/maxlcummins/pipelord [48]. Scripts used to process and visualize data are available on Github (https://www.github.com/maxlcummins/APG-OHEC-Retro-M2). Default parameters were used unless otherwise stated.

### Genome pre-processing and quality control

Read preprocessing was performed using fastp [24] v0.20.1 and genome assembly with Spades [49] v3.14 via shovill (https://www.github.com/tseeman/shovill) v1.0.4. Quality control procedures undertaken are described in our recent publication [46]. In short, we required (i) assembly sizes to fall within the expected range for *E. coli* as per the RefSeq database, (ii) all seven *E. coli* (Achtman scheme) multilocus sequence type (MLST) alleles to be detected, (iii) the majority of sequence reads to map to *E. coli* (Kraken2 and Bracken), (iv) CheckM2 contamination scores below 10% and (v) CheckM2 completeness scores above 90%. Isolates were also required to have 95% of 2,513 core-genome multilocus sequence type (cgMLST) alleles present to be considered for further analysis.

### Phylogenetic analysis

cgMLSTs were generated using chewBBACA [50] v2.8.5 (cgMLST schema https://enterobase.warwick.ac.uk/schemes/Escherichia.cgMLSTv1 – accessed 19 July 2022) via Coreugate (https://www.github.com/MDU-PHL/Coreugate) v2.0.5 as were the pairwise cgMLST allelic distances which were used to generate a phylogenetic tree using rapidNJ [51] v2.3.3. Phylogroups were assigned using Clermontyper [52] v2.0.3.

### Genotyping

Carriage of genes associated with AMR, virulence and plasmids was determined using Abricate (https://www.github.com/tseemann/abricate) v1.0.1 (≥90% length and ≥90% identity). Nucleotide databases included virulence factor database [53], ISFinder [54] and PlasmidFinder [55] as well as a custom database, all of which are available at https://www.github.com/maxlcummins/APG-OHEC-Retro-M2. We also performed pMLST using pmlst [55], including RSTs. Note that RST profiles are defined based on allelic combinations of the FIA, FIB, FII and FIC replicons, providing finer resolution of diversity among F plasmids. By convention, FIA, FIB, FII and FIC are represented in the format Fx:Ay:Bz, where the numbers denote the specific alleles of the repFII, repFIA and repFIB loci, respectively (e.g. F1:A2:B10); repFIC (C) is typically omitted unless it is detected.

Genomes carrying genes associated with resistance to three or more classes of antimicrobials were considered MDR, while those which carried genes associated with resistance to one or more classes were deemed as the highest priority by the WHO as resistant to critically important antimicrobials (including third-generation cephalosporins, carbapenems, fluoroquinolones, macrolides and colistin) [56]. We used a modified Liu *et al*. criteria [57] to determine ColVLP carriage as previously described [9]. A genome with carriage of one or more ColV genes (defined as ≥95% nucleotide identity and ≥90% coverage) from four or more of the following sets of genes was considered to carry a ColVLP: (i) *cvaABC* and *cvi* (the ColV operon), (ii) *iroBCDEN* (salmochelin operon), (iii) *iucABCD* and *iutA* (the aerobactin operon), (iv) *etsABC*, (v) *ompT* and *hylF* and (vi) *sitABCD*. Scripts used to determine ColVLP carriage and process RST data are available at https://www.github.com/maxlcummins/APG-OHEC-Retro-M2.

### Plasmid clustering and reference plasmid analyses

Putative plasmid identification and plasmid clustering were performed using MOB-suite v3.1.0 [58] with default parameters. MOB-suite screens genomic assemblies for replicon and relaxase markers, removes putative chromosomal contigs and those enriched in repeat regions, aggregates putative plasmid contigs and assigns them into plasmid clusters following alignment to a reference database of 23,700 curated closed plasmid sequences. Putative plasmids were required to have ≥60% non-overlapping, aggregated coverage and ≥80% nucleotide identity to reference plasmids, and multi-copy plasmids within a genome were counted only once.

To explore the potential biological relevance of the plasmid clusters identified, we genotyped the MOB-suite reference database comprising 23,700 reference plasmids to assess their predominant pMLST profiles, virulence and resistance traits. This analysis was also performed as described above using pipelord. This data is available in Table S2 at https://www.github.com/maxlcummins/APG-OHEC-Retro-M2, as is the specific version of the reference plasmid database.

Plasmid alignments were performed using Proksee [59], with reference plasmids and representative genomes of diverse ST selected using random sampling using R in the code provided at https://www.github.com/maxlcummins/APG-OHEC-Retro-M2. Default settings for Proksee were utilized; annotations were manually coloured to allow highlighting of virulence-associated genes (VAGs) and ARGs.

### Statistical analysis

Statistical analyses were performed using R v4.0.2. Packages utilized for general processing and visualization in R include ggvenn [60] v0.1.10, igraph v2.0.3 [61], tidyverse [62] v2.0.0, ggplot2 [63] v3.4.4, ggrepel [64] v0.9.3, ggalluvial [65] v0.12.5, caret [66] v6.0-94, pheatmap [67] v1.0.12, circlize [68] v0.4.15, plotly [69] v4.10.2, tidyverse [62] v2.0.0 and scales [70] v1.3.0. We also computed Shannon entropy scores to measure the distribution of plasmid clusters throughout phylogenetic backgrounds (specifically phylogroups), similar to Robertson *et al*. [71]; we computed ours in R using the formula ‘S = -Σ i *Pi* ln*Pi*’ where *Pi* is the probability of a plasmid cluster occurring in a given phylogroup. Because natural logarithms were used, entropy values are expressed in nats (natural units of information; 1 nat=1/ln 2 ≈ 1.44 bits).

## Results

### Australian *E. coli* are phylogenetically diverse

**Fig. 1.**
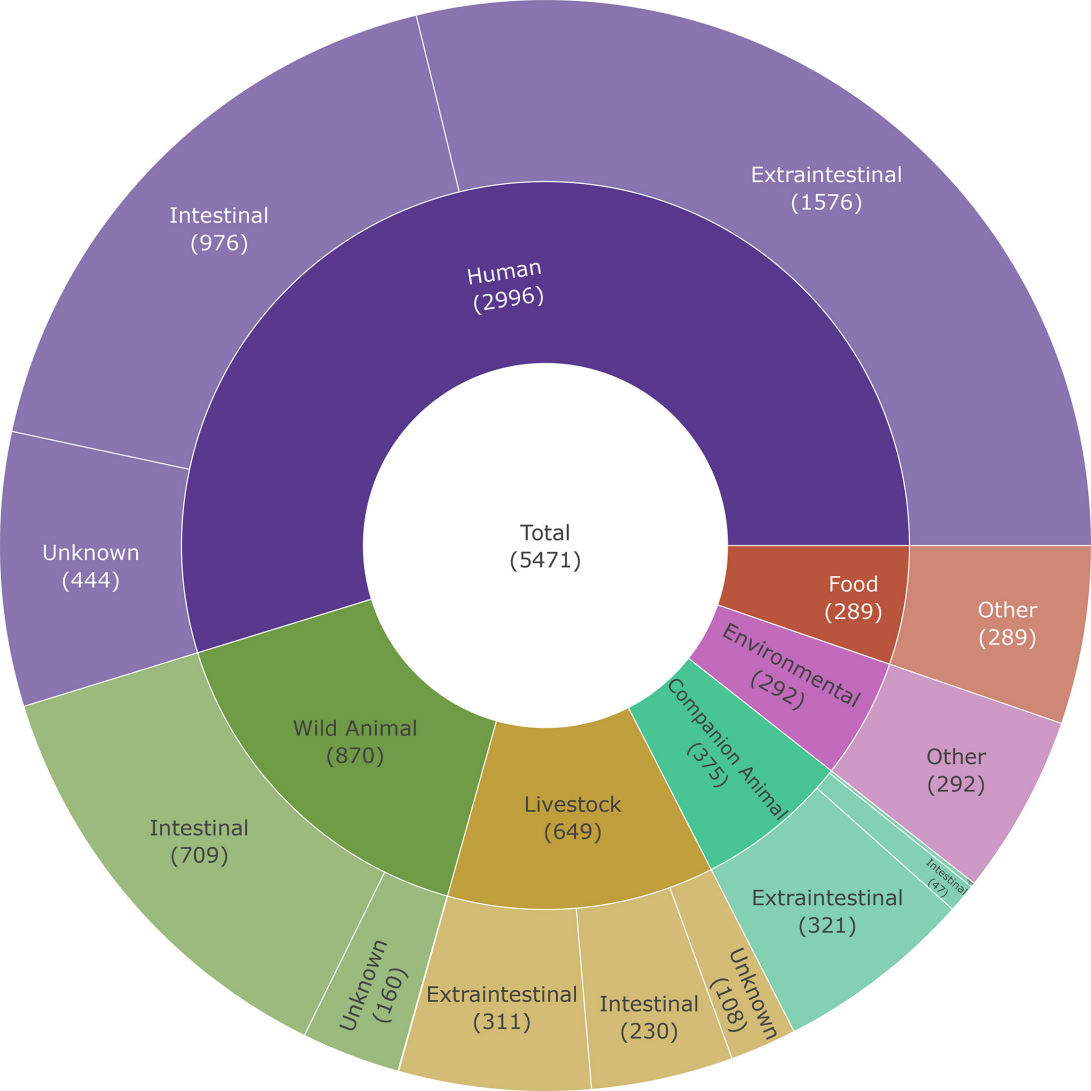
Collection composition. This sunburst chart visualizes the number and proportion of strains among a given source and intestinal/extraintestinal niche. Note: A total of seven additional companion animal strains were unable to be categorized as either intestinal or extraintestinal due to missing metadata.

While phylogroup B2 was most commonly identified (*n*=2,175/5,471; 39.8%), all eight *sensu stricto* phylogroups (i.e. A, B1, B2, C, D, E, F and G) were detected (Table 1). Additionally, 16/5,471 genomes were found to be members of clades I, III, IV, ‘E or clade I’ or designated as ‘Unknown’, due to their atypical genotypic profiles relative to the loci utilized in the phylogrouping scheme. Given the infrequency of the latter groups, phylogroup level comparisons will include only genomes from phylogroups A–G. The most common source associated with each phylogroup was humans, likely attributable to the frequency of genomes from this source (Fig. 1), except for phylogroups B1 (predominantly from wild animals), E and G (each predominantly from livestock).

**Table 1.** Phylogroup representation among genomes under investigation by source

Phylogroup	Companion animal	Environmental	Food	Human	Livestock	Wild animal	Total	Percentage (%)
**A**	20	68	66	346	231	224	955	17.50
**B1**	52	120	54	178	84	213	701	12.80
**B2**	231	39	37	1,637	77	154	2,175	39.80
**C**	13	2	4	89	6	10	124	2.30
**D**	29	29	60	481	54	139	792	14.50
**E**	4	16	30	48	89	19	206	3.80
**E or clade I**	0	0	2	0	2	0	4	0.10
**F**	14	10	17	194	4	109	348	6.40
**G**	8	8	17	19	101	1	154	2.80
**Unknown**	0	0	2	1	0	0	3	0.10
**Clade I**	2	0	0	3	1	0	6	0.10
**Clade III**	2	0	0	0	0	0	2	0.00
**Clade IV**	0	0	0	0	0	1	1	0.00

A total of 838 STs were identified [Table S1 (https://www.github.com/maxlcummins/APG-OHEC-Retro-M2)]. Among the most common STs identified were major ExPEC lineages including ST131 [phylogroup B2; 15.4% (*n*=841/5,471)], ST95 [phylogroup B2; 4.5% (*n*=247/5,471)], ST10 [phylogroup A; 4.1% (*n*=223/5,471)], ST1193 [phylogroup B2; 3.7% (*n*=201/5,471)] and ST73 [phylogroup B2; 3.3% (*n*=183/5,471)]. Cumulatively, these STs comprised 31.0% (*n*=1,695/5,471), singleton STs comprised 8.7% (475/5,471) and STs with between 2 and 174 representatives comprised the remaining 60.0% (3,280/5,471). See Table S1 (https://www.github.com/maxlcummins/APG-OHEC-Retro-M2) for ST data for all genomes under analysis.

### Non-human strains disproportionately carry novel plasmid clusters

MOB-suite identified a total of 22,534 putative plasmids (see Methods for details), of which 21,814 (96.8%) originated from 547 known primary plasmid clusters (hereon referred to as plasmid clusters, all of which exhibit pairwise mash distances ≤0.05). The remaining 720/22,534 (3.2%) putative plasmids did not match any previously designated clusters (Table 2). The 20 most frequent clusters comprised 41.3% (*n*=9,308/22,534) of putative plasmids within the collection. Detected putative plasmids were highly diverse, carrying a total of 92 rep genes, including 13 Col-type, 27 typeable and 55 non-typeable replicase clusters and exhibiting a diversity of sizes [median=16 kb; interquartile range (IQR)=4.2–74.8 kb; range=1–436.7 kb], the distribution of which can be seen in Fig. S1.

**Table 2.** Source distribution of the most common plasmid clusters identified within the collection Columns also detail the predominant replicon among these plasmid clusters and the most frequent RST identified among genomes carrying a putative plasmid such as a plasmid cluster, where the predominant replicon was an F replicon.

Primary cluster ID	Predominant replicon	RST	Human	Livestock	Wild animal	Food	Companion animal	Environ -mental	Total	Percentage (%)
**AB685**	Col(MG828)	–	610	210	71	89	41	4	1,025	4.55
**AA579**	rep_cluster_1778	–	759	62	121	45	26	8	1,021	4.53
**AA977**	Col156	–	732	0	49	1	24	2	808	3.59
**AA474**	I-gamma/K1	–	388	95	203	42	35	19	782	3.47
**AA171**	FIA	F1:A2:B20	545	0	33	0	22	1	601	2.67
**AG884**	–	–	248	39	101	114	3	67	572	2.54
**AA176**	FIB	F18:A-:B1	180	207	51	80	33	6	557	2.47
**AC748**	Col(BS512)	–	487	18	26	1	16	3	551	2.45
**AA337**	FIA	F29:A-:B10	344	0	48	0	32	2	426	1.89
**AA378**	HI1B	–	162	81	78	50	17	18	406	1.80
**AA352**	rep_cluster_2131	–	283	0	18	0	6	0	307	1.36
**AA282**	K2/Z	–	265	2	21	0	13	1	302	1.34
**AA336**	FIA	F2:A-:B-	235	35	19	0	11	2	302	1.34
**AA747**	rep_cluster_488	–	138	62	40	28	10	24	302	1.34
**AC509**	rep_cluster_2350	–	152	73	11	28	4	2	270	1.20
**AF232**	–	–	66	28	60	20	9	58	241	1.07
**AG294**	–	–	97	23	45	43	0	21	229	1.02
**AA979**	Col156	–	42	74	61	9	9	8	203	0.90
**AB686**	Col(MG828)	–	98	40	16	19	27	2	202	0.90
**AA174**	FII	F1:A1:B1	146	4	19	20	11	1	201	0.89
**Novel**	FIA	F-:A-:B-	286	120	168	72	10	64	720	3.20
**Other**	–	–	6,187	2,205	2,118	1,131	437	428	12,506	55.50
**Total**	na	–	12,450	3,378	3,377	1,792	796	741	22,534	100.00

**Fig. 2.**
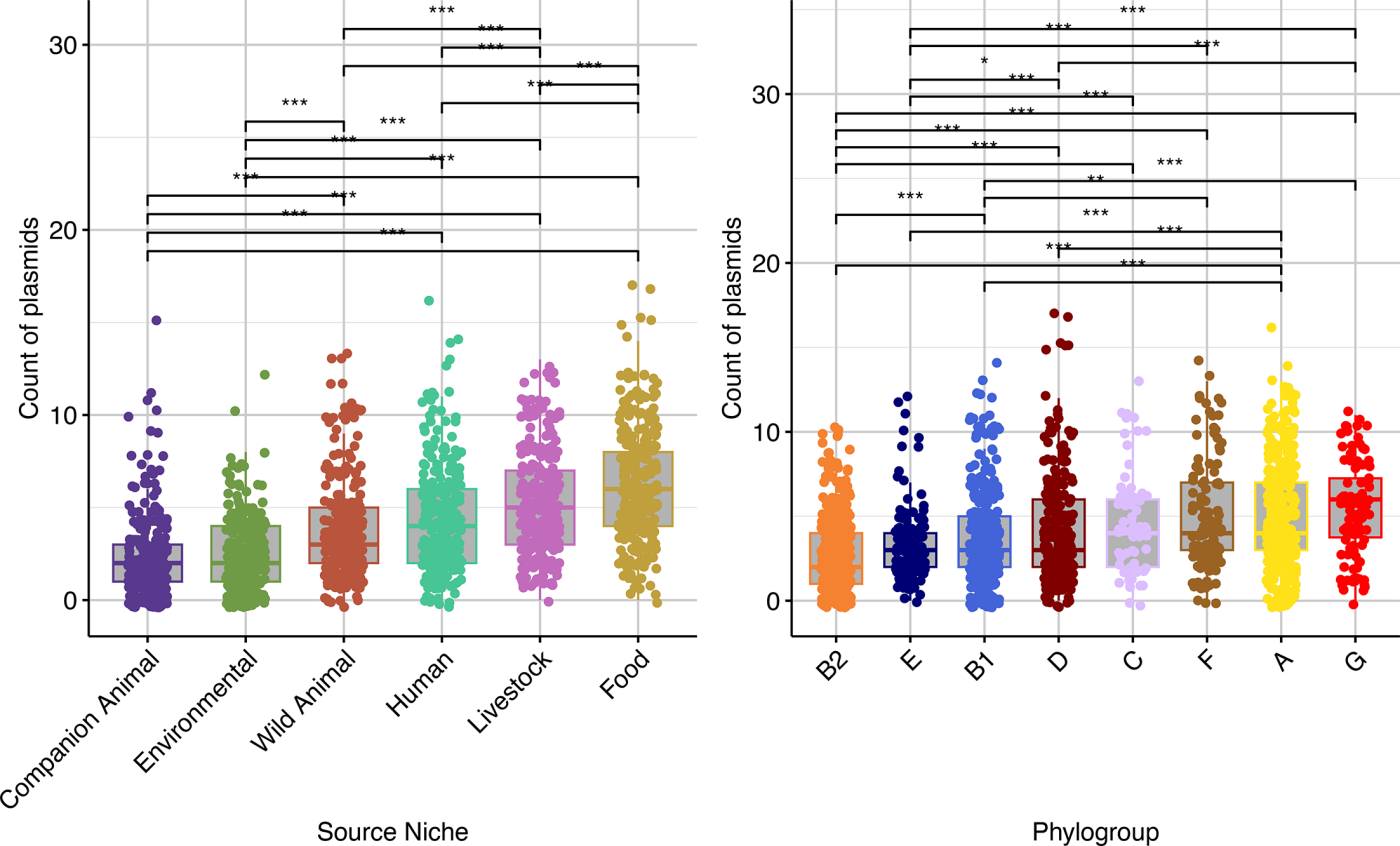
Boxplots show *putative plasmid* counts among strains by source and phylogroup. Boxes represent the IQR with the median indicated by a horizontal line; whiskers extend to 1.5× IQR, and points beyond the whiskers denote outliers. Statistically significant differences between group means are denoted with asterisks (***P<0.001, **P<0.01, *P<0.05), as determined using Wilcoxon tests.

Strains varied significantly in their mean number of putative plasmids based on their source of isolation (Fig. 2), while controlling for phylogroup membership (Analysis of Covariance [ANCOVA] *P*-value: <0.05). Strains from companion animals (*µ*=2.21) and environmental sources (*µ*=2.82) had significantly fewer putative plasmids than those from other sources (*P*-values <0.05), while those from food had significantly more than all other sources (*µ*=6.3, *P*-values <0.05). Similarly, we found that strains differed significantly in their count of putative plasmids carried according to their phylogroup while controlling for source of isolation (ANCOVA *P*-value <2e−16); strains of phylogroups B1, B2, D and E generally carried fewer plasmids (*µ* 3.80, 2.94, 4.20 and 3.19, respectively) while those from phylogroups A, C, F and G carried more plasmids (*µ* 5.11, 4.67, 4.83 and 5.44, respectively).

While human and wild animal sourced strains carried the majority of putative plasmids which did not cluster with known plasmid clusters (hereon referred to as novel putative plasmids), when adjusting for the number of strains per source (defined by the ratio of total plasmids identified within a source to the total genomes originating from a source), non-human sources generally carried novel putative plasmids more frequently. For example, while 8.4% (*n*=286/2,996) of human-sourced strains carried novel putative plasmids (*µ*=0.095), those from livestock (*µ*=0.18; *n*=120/649; 18.4%), wild animals (*µ*=0.19; *n*=160/870; 18.4%), food (*µ*=0.25; *n*=72/289; 24.2%) and the environment (*µ*=0.22; *n*=64/292; 21.9%) often exhibited carriage of such plasmids twice as frequently. Notably, strains sourced from companion animals carried novel putative plasmids the least frequently, with only 2.7% (*µ*=0.027; *n*=10/375) of such strains carrying novel putative plasmids.

### F virulence plasmids are prevalent in Australian *E. coli*

A genotypic analysis of all reference plasmids within the MOB-suite database was undertaken. Note that this manuscript refers to ‘plasmid constructs’ detected by MOB-suite within our 5,471 *E. coli* genomes as ‘putative plasmids’, to primary plasmid clusters from MOB-suite (e.g. AA337, AA171, etc.) as ‘plasmid clusters’ and to complete plasmid sequences from the MOB-suite database as ‘reference plasmids’.

Reference plasmid analyses were performed to assess the carriage of VAGs, ARGs and plasmid multilocus sequence types (pMLSTs) of reference plasmids from the plasmid clusters identified within our cohort. We classified 152/547 (27.8%) plasmid clusters as ARG-associated (when more than half of their representatives carried ≥1 ARG). Similarly, we classified 60/547 (11.0%) as VAG-associated clusters (when more than half of their representatives carried ≥1 VAG). We also identified 18/547 plasmids which met both such criteria. Among plasmid clusters associated with virulence loci, F replicons predominated (Fig. S2). As such, we focused our analysis on plasmid clusters linked with F replicons, hereon referred to as F plasmid clusters, given the known roles of such plasmids in mediating virulence and resistance dissemination. Additionally, we computed Shannon’s entropy for each plasmid cluster based on the distribution of its associated putative plasmids across *E. coli* phylogroups within our genomic dataset. These scores quantify the extent to which plasmid clusters are distributed among, and shared between, different host phylogenetic backgrounds (Table 3).

**Table 3.** Characteristics of common F plasmid clusters and their source distribution among Australian *E. coli* Screening of genomes under investigation determined whether strains carried ColV plasmids determined as such using the Liu criteria (see Methods) or senB-associated plasmids determined based on carriage of senB; plasmids classified as ‘Other’ met neither criterion. RSTs shown are those which were most numerous among genomes carrying a putative plasmid of a given cluster. Phylogroup entropy is a metric reflecting plasmid promiscuity where a higher value indicates an increased spread throughout a greater diversity of phylogroups. Columns four to nine detail the proportion of genomes carrying a given plasmid cluster with a particular genomic trait (see Methods). HPI, high-pathogenicity island; CIA, critically important antibiotic resistance. The final seven columns detail counts of a given plasmid cluster relative to a particular source: H, human; L, livestock; WA, wild animal; F, food; E, environmental; CA, companion animal.

Plasmid cluster	RST	Phylogroup entropy	ColV+* (%)	*senB*+* (%)	HPI*	CIA*	MDR*	intI1*	H	L	WA	F	E	CA	Plasmid cluster (count)
AA171	F1:A2:B20	0.741	15 (2.5%)	422 (70.2%)	590 (98.2%)	585 (97.3%)	518 (86.2%)	30 (5.0%)	545 (90.7%)	0 (0.0%)	33 (5.5%)	0 (0.0%)	1 (0.2%)	22 (3.7%)	601
AA176	F18:A-:B1	1.837	541 (97.1%)	0 (0.0%)	330 (59.2%)	126 (22.6%)	269 (48.3%)	267 (47.9%)	180 (32.3%)	207 (37.2%)	51 (9.2%)	80 (14.4%)	6 (1.1%)	33 (5.9%)	557
AA337	F29:A-:B10	0.695	19 (4.5%)	386 (90.6%)	425 (99.8%)	273 (64.1%)	172 (40.4%)	50 (11.7%)	344 (80.8%)	0 (0.0%)	48 (11.3%)	0 (0.0%)	2 (0.5%)	32 (7.5%)	426
AA352	F-:A1:B10	0.711	3 (1.0%)	265 (86.3%)	302 (98.4%)	281 (91.5%)	267 (87.0%)	77 (25.1%)	283 (92.2%)	0 (0.0%)	18 (5.9%)	0 (0.0%)	0 (0.0%)	6 (2.0%)	307
AA174	F1:A1:B1	0.757	14 (7.0%)	12 (6.0%)	149 (74.1%)	174 (86.6%)	179 (89.1%)	59 (29.4%)	146 (72.6%)	4 (2.0%)	19 (9.5%)	20 (10.0%)	1 (0.5%)	11 (5.5%)	201
AA324	F36!/F31!:A4:B1	0.627	2 (1.0%)	76 (38.8%)	183 (93.4%)	196 (100.0%)	191 (97.4%)	121 (61.7%)	176 (89.8%)	0 (0.0%)	16 (8.2%)	0 (0.0%)	1 (0.5%)	3 (1.5%)	196
AA175	F24:A-:B40	1.074	162 (88.5%)	2 (1.1%)	69 (37.7%)	57 (31.1%)	71 (38.8%)	56 (30.6%)	32 (17.5%)	52 (28.4%)	38 (20.8%)	47 (25.7%)	4 (2.2%)	10 (5.5%)	183
AA297	F51:A-:B10	0.585	46 (28.2%)	153 (93.9%)	158 (96.9%)	36 (22.1%)	119 (73.0%)	110 (67.5%)	147 (90.2%)	1 (0.6%)	5 (3.1%)	0 (0.0%)	0 (0.0%)	10 (6.1%)	163
AA315	F24:A-:B1	1.377	16 (10.7%)	3 (2.0%)	38 (25.3%)	54 (36.0%)	51 (34.0%)	27 (18.0%)	22 (14.7%)	29 (19.3%)	41 (27.3%)	32 (21.3%)	18 (12.0%)	8 (5.3%)	150
AE638	F18:A-:B1	0.747	73 (50.0%)	19 (13.0%)	100 (68.5%)	37 (25.3%)	39 (26.7%)	29 (19.9%)	51 (34.9%)	30 (20.5%)	25 (17.1%)	13 (8.9%)	5 (3.4%)	22 (15.1%)	146
AA179	F18:A5:B1	1.279	94 (65.3%)	10 (6.9%)	74 (51.4%)	83 (57.6%)	82 (56.9%)	39 (27.1%)	50 (34.2%)	32 (21.9%)	28 (19.2%)	19 (13%)	2 (1.4%)	13 (8.9%)	144
AA735	F2:A1:B-	0.401	1 (0.8%)	11 (9.2%)	114 (95.8%)	118 (99.2%)	113 (95.0%)	67 (56.3%)	111 (76%)	0 (0%)	7 (4.8%)	0 (0%)	0 (0%)	1 (0.7%)	119

*Traits are present with whole-genome sequence data for a given strain. See Fig. S3 for ARG and VAG carriage and Table S2 (https://github.com/maxlcummins/APG-OHEC-Retro-M2) for overall genotypic characteristics of these and other reference plasmids (as per analysis of the reference plasmid database).

### ColV- and *senB*-associated virulence plasmids exhibit different phylogenetic and source distributions

Among the 12 most frequent virulence-associated F plasmid clusters, ColV- and *senB*-associated clusters predominated (Table 2). These were observed in 2,374/5,471 (42.6%) of genomes; 1,047/5,471 (19.1%) genomes met criteria for ColV-like plasmid carriage, while 1,327/5,471 (24.3%) carried *senB* [Table S1 (https://www.github.com/maxlcummins/APG-OHEC-Retro-M2)]. Additionally, 64/5,471 strains met both criteria, of which 48/64 (75.0%) were ST73, an ST known for carriage of *senB*-associated plasmids and chromosomal genomic islands housing VAGs, typically iron acquisition operons, that are associated with ColV-like plasmids [17]. Additionally, *senB*-associated plasmid clusters exhibited greater source restriction, being primarily associated with human hosts, unlike ColV-associated plasmid clusters which were spread across sources (Table 3).

**Fig. 3.**
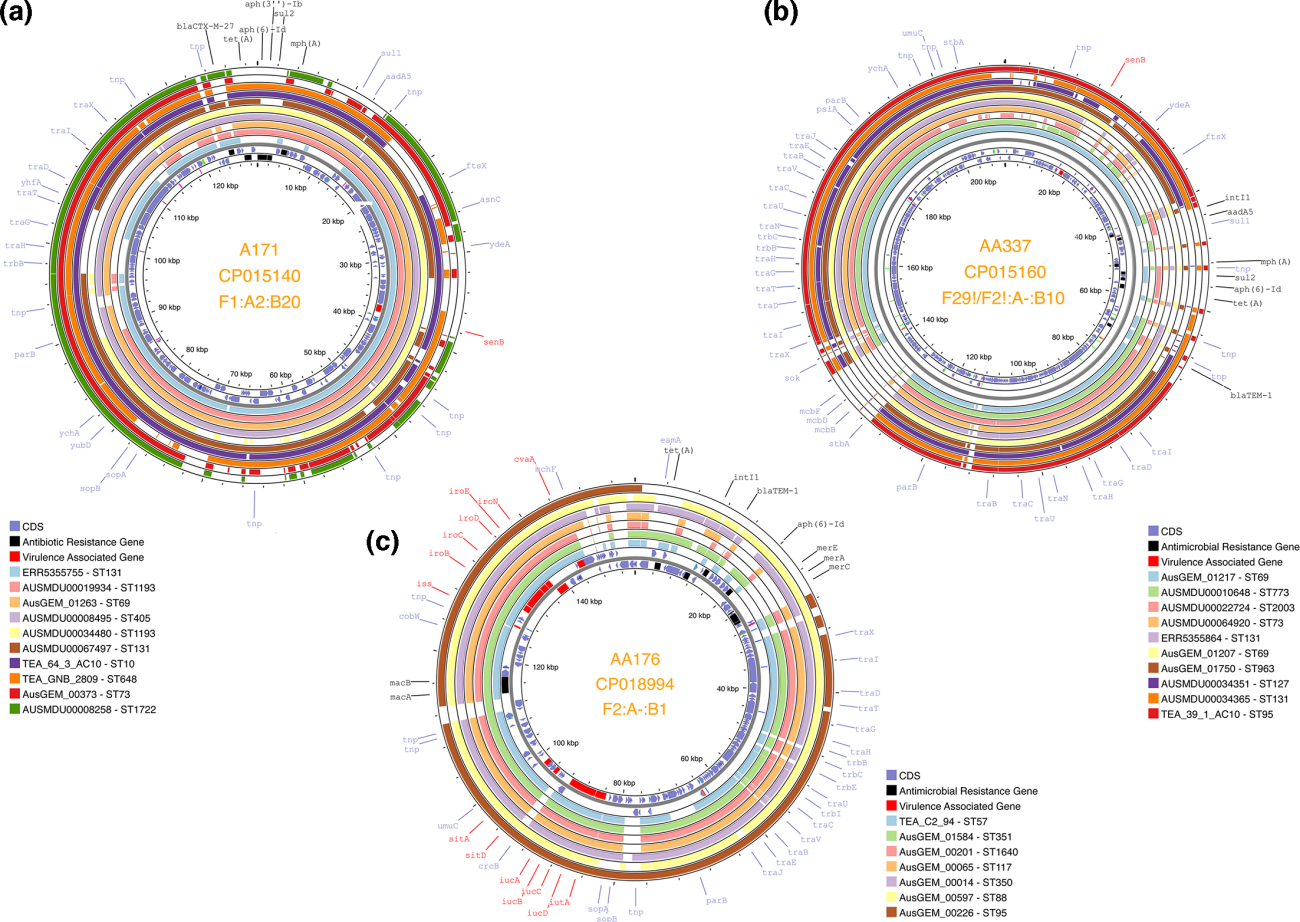
Plasmid alignments of major F plasmid clusters. This figure visualizes the sequence coverage of reference plasmids from the three primary plasmid clusters identified within the current cohort (a - A171, b - AA337, and c - AA176). The innermost band indicates base-pair coordinates, the next two bands display the strandedness and approximate size of coding sequences and the subsequent coloured rings indicate where a given stretch of reference sequence is present within the corresponding genome (as per the legend corresponding to each plasmid map). The outermost annotations highlight the gene name associated with a given coding sequence. Virulence genes are coloured in red, while ARGs are coloured in black. All other genes, including those with no known name or function, are shown in blue.

Three primary F plasmid clusters were identified, AA171, AA176 and AA337 (Table 3). Genomes which carried putative plasmids from these plasmid clusters were identified, and their assemblies were aligned to corresponding reference plasmids (see methods). This highlighted extensive conservation of reference plasmid backbone as well as VAGs, but limited alignment of antibiotic resistance regions associated with these reference plasmids, even across diverse STs (Fig. 3). Putative plasmids from plasmid cluster AA171 are structurally similar to pEC732_2 of RST F1:A2:B20 (Fig. 3), a plasmid closely related to pG150_1 and pCA08 [72]. They were identified in 601 samples that were predominantly of human origin (*n*=545/601; 90.7%). ST131 (*n*=444/601; 73.9%) strains dominated those carrying putative plasmids from this plasmid cluster, but other STs including ST1193 (*n*=35/601; 5.8%), ST69 (*n*=19/601; 3.2%), ST405 (*n*=16/601; 2.6%), ST450 (*n*=13/601; 2.2%), ST73 (*n*=12/601; 2.0%) and even strains of phylogroup A ST10 (*n*=10/601; 1.7%) [Table S1 (https://www.github.com/maxlcummins/APG-OHEC-Retro-M2)] were also observed. This plasmid cluster’s spread was limited throughout other phylogenetic backgrounds, as indicated by its low phylogroup entropy score [0.741 nats (see Methods)]. Among reference plasmids from this plasmid cluster, 28/41 (68.3%) carried critically important ARGs (Fig. S3). Critically important antibiotic (CIA) resistance was near ubiquitous in *E. coli* that carry putative plasmids belonging to the plasmid cluster AA171 (*n*=585/601; 97.3%), and most were MDR (*n*=518/601; 86.2%). However, carriage of a class 1 integron integrase gene (*intI1*) was rare (*n*=30/601; 5.0%).

In total, 557 genomes representing diverse STs including ST95 (*n*=118/557; 21.2%), ST117 (*n*=99/557; 17.8%) and ST57 (*n*=53/557; 9.5%) but also detected among ST58 (*n*=22/557; 4.0%), ST88 (*n*=21/557; 3.8%), ST350 (*n*=15/557; 2.7%), ST93 (*n*=12/557; 2.2%) and ST131 (*n*=10/557; 1.8%) were determined to carry putative plasmids from plasmid cluster AA176. These are structurally similar to pECAZ147_1, which has an RST of F2:A-:B1 and is a close relative of pCERC4, pCERC5 and pCERC6 [32]. Putative plasmids belonging to this plasmid cluster were identified across eight *sensu stricto* phylogroups (A–G) and in all sources analysed but especially livestock (*n*=207/557; 37.2%), humans (*n*=180/557; 32.3%) and food (*n*=80/557; 14.4%). Among the 287 livestock and food samples carrying these putative plasmids, most were sourced from poultry (*n*=172/287; 59.9%) and poultry meat (*n*=80/287; 27.9%), with a small number also sourced from swine (*n*=35/287; 12.2%).

Among the 67 representatives of plasmid cluster AA176 in the MOB-suite database, critically important ARGs (i.e. those conferring resistance to third-generation cephalosporins, carbapenems, fluoroquinolones, macrolides and colistin) were detected (*n*=12/67; 17.9%) (Fig. S3). A similar proportion of genomes (*n*=126/557; 22.6%) under analysis carrying putative plasmids from this plasmid cluster was also found to carry critically important ARGs (Table 3), while genotypic multidrug resistance and carriage of class 1 integrase gene *intI1* was observed in almost half of such genomes (*n*=269/557; 48.3% and *n*=267/557; 47.9%, respectively). Reference plasmids from this cluster also frequently carried VAGs involved in iron acquisition such as *sitA*, *iucABCD*/*iutA* (aerobactin) and *iroBCDEN* (salmochelin), as well as the protectin *iss* (increased serum survival) and *ompT* (outer membrane protein) (Fig. S3). Among the 67 representatives in the reference plasmid database, RSTs included F18:A-:B1 (*n*=23/67; 34.0%), F2:A-:B1 (*n*=12/67; 17.9%) and F24:A-:B1 (*n*=11/67; 16.4%). We also found that putative plasmids from this plasmid cluster exhibited a phylogroup entropy of 1.837 nats – the highest among all virulence-associated F plasmids under analysis, indicating its capacity for carriage among broad phylogenetic backgrounds.

AA337 was the third most common F plasmid cluster in our collection (*n*=426/5,471; 7.8%), and the eighth most common plasmid cluster identified overall. Putative plasmids from this plasmid cluster are structurally similar to pECO-fce (RST F29!F2!:A-:B10). (Fig. 3), which is closely related to pUTI89, RS218 and pEC14_114 [73]. Reference plasmids of AA337 are predominantly of RST F29:A-:B10 (*n*=19/23; 82.6%) and frequently carry *senB* (Table 3). A diversity of RSTs was observed (46 in total); however, the majority (*n*=338/426; 77.7%) were F29:A-:B10. CIA resistance-associated genes were observed in just 3/23 (13.0%) AA337 reference plasmids (Fig. S3). In contrast, among genomes under analysis carrying putative plasmids from this plasmid cluster, 243/426 (64.1%) exhibited carriage of critically important ARGs, indicating that such loci in such strains may be carried either chromosomally or on other mobile genetic elements. While *intI1* carriage in such genomes was infrequent (*n*=50/426; 11.7%), genotypic multidrug resistance was frequently observed (*n*=172/426; 40.4%). Among the *E. coli* carrying putative plasmids from plasmid cluster AA337, phylogroup B2 (*n*=267/426; 62.3%), including ST131 (*n*=102/426; 23.5%), ST95 (*n*=54/426; 17.2%), ST127 and ST73 (both *n*=31/426; 7.1%) and phylogroup D (*n*=141/426; 32.0%) predominated. Among the phylogroup D isolates, ST963 (*n*=75/426; 17.6%) and ST69 (*n*=43/426; 10.1%) were well represented. The majority were sourced from humans (*n*=344/426; 80.1%) with the remainder from wild animals (*n*=48/426; 11.3%), companion animals (*n*=32/426; 7.5%) and the environment (*n*=2/426; 0.5%); no samples from food or livestock were found to carry putative plasmid cluster from plasmid cluster AA337. Similar to putative plasmids from plasmid cluster AA171, those from AA337 exhibited a low phylogroup entropy score (0.695 nats), highlighting its relative restriction throughout phylogenetic backgrounds.

### ColV and *senB*-associated plasmids are present in clonally related strains isolated from different sources

We calculated the cgMLST allelic distance of strains in the collection and identified 32.6% (*n*=1,782/5,471) that differed by ≤10 cgMLST alleles. These strains formed a total of 11,568 pairwise combinations, with each strain differing by 10 or fewer cgMLST alleles to 1 or more other strains. Of these, 790/11,568 (6.8%) pairs (comprising 191 isolates) were collected from different sources (hereon referred to as cross-source linkages), while 10,778/11,568 (93.2%) pairs (comprising 1743 isolates) were found to share a source. Among strain pairs originating from different sources, we sought to identify which putative plasmids from F plasmid clusters were present. We found that of the 628 pairs of strains meeting this criteria, a total of 560/628 (89.2%) carried putative plasmids belonging to the same F plasmid cluster.

**Fig. 4.**
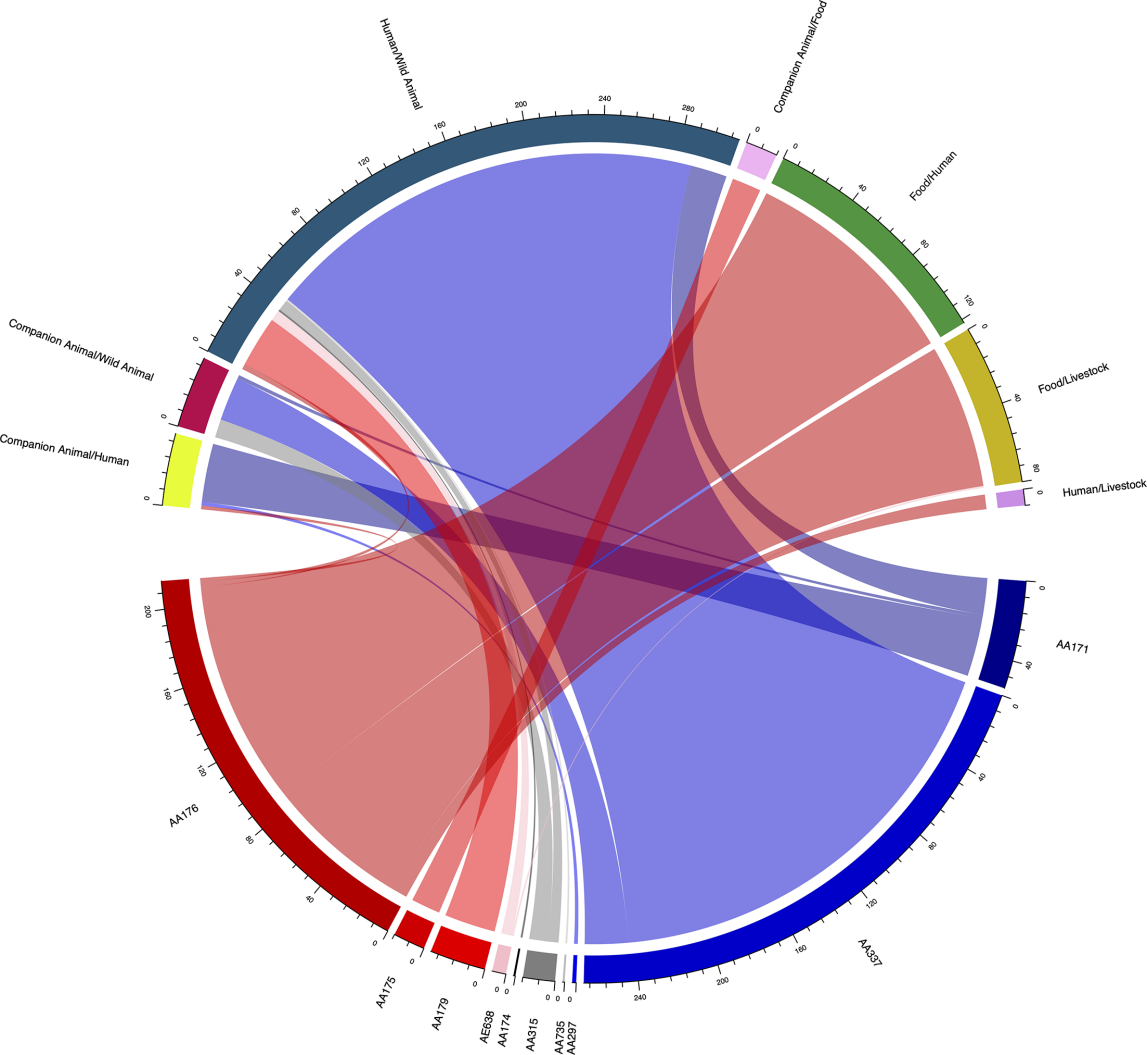
F plasmid cluster carriage among inter-source strain pairs exhibiting close phylogenetic overlap (≤10 allelic differences). This figure visualizes ten F plasmid clusters and their association with combinations of sources from which pairs of strains exhibit close phylogenetic overlap. Numbers around the arcs indicate the number of strain pairs carrying a putative plasmid from each plasmid cluster (bottom) and the number of strain pairs from a given source combination (top). Sections in red and pink (clusters AA176, AA175, AA179 and AE638) are ColV-associated plasmid clusters, and those in blue (AA297, AA337 and AA171) are senB-associated plasmid clusters, while those in shades of grey (AA174, AA315 and AA735) infrequently meet criteria for ColV-like plasmids and infrequently carry senB.

Cumulatively, these putative plasmids were represented by a total of ten F plasmid clusters (Fig. 4). Different plasmid types implicated in these cross-source linkages exhibited association with particular source combinations. Most commonly implicated were putative plasmids of plasmid cluster AA176, with a total of 206 cross-source linkages. Of these, most linkages occurred between strains spanning food and human sources (*n*=122/206; 61.0%) as well as food and livestock sources (*n*=79/206; 33.3%), with a small number of pairs also linking strains from humans and livestock (*n*=8/318; 5.0%), wild animals and human (3/206; 1.5%) and companion animals and humans (*n*=2/318; 0.6%). Plasmid cluster AA337 was also commonly implicated in cross-source linkages (*n*=267); however, it was associated predominantly with human and wild animal linkages (*n*=243/267; 93.5%), with a small number of linkages also detected between companion animals and wild animals (*n*=24/267; 6.5%). Cumulatively, these two clusters represented 82.5% (*n*=473/606) of putative F plasmids among pairs of strains implicated in cross-source linkage. Notably, putative plasmids from plasmid cluster AA171 were relatively infrequently detected in strain pairs originating from different sources despite their high prevalence in the collection overall, being observed in only 8.0% (*n*=54/606) of cross-source matching pairs, of which 38.5% (*n*=20/52) were links between humans and wild animals, 61.5% (*n*=32/52) between companion animals and humans and 3.8% (*n*=2/52) were in links between companion animals and wild animals.

## Discussion

High rates of F plasmid carriage are a feature of *E. coli* causing BSI [30,42, 45] and UTI [17]. In the current study, we sought to expand on these findings by examining the carriage of F plasmids and their important virulence gene cargo that circulates within a large collection of 5,471 Australian *E. coli* whose core genomes were recently characterized [46]. The *E. coli* cohort provided a unique opportunity to characterize F plasmids carried by *E. coli* lineages from disparate sources. Specifically, we linked important ExPEC virulence gene cargo with specific putative F plasmid clusters which are known to mediate ExPEC disease outcomes in humans and food animals and others primarily in humans.

### Two F plasmid lineages are dominant in ExPEC disease

F plasmids are important in ExPEC disease [30,42]. Here, we identified three dominant plasmid clusters associated with virulence gene carriage in our expansive Australian *E. coli* collection: a ColV-associated AA176 plasmid cluster and two *cjrABC-senB*-associated plasmid clusters, AA171 and AA337. The prominence of these plasmids reinforces their central role in ExPEC virulence, consistent with previous studies linking ColV-associated and *cjrABC-senB*-associated F plasmids to BSI and UTI [9,30]. Importantly, our study also sheds light on the One Health context of such plasmids.

Putative plasmids belonging to the ColV-associated AA176 plasmid cluster displayed the broadest host and phylogroup range, being identified in humans, livestock, wildlife, companion animal and food sources, but were especially prevalent in poultry. This mirrors earlier reports describing ColV plasmids as defining features of avian pathogenic *E. coli* which are frequently recovered from diseased poultry and retail meat [74,77]. Our data indicates their capacity to be stably maintained across a greater breadth of phylogenetic backgrounds and hosts; observations are in accordance with our earlier study of 34,176 *E. coli* genomes from public repositories [9,12, 78].

In contrast, *senB*-associated putative plasmids from plasmid clusters AA171 and AA337 showed a more restricted host range and were not observed in isolates from livestock, but rather were frequently observed in humans, companion animals and wildlife. Our previous large-scale *E. coli* analysis is also in support of this observation [9]. Such host-specific segregation could reflect ecological or physiological constraints that limit their maintenance in livestock-associated lineages; however, further functional studies would be required to test a potential fitness cost of *cjrABC-senB*-associated plasmids in such hosts.

Despite the differences in source segregation among these groups of plasmids, it is critical to note that both are strongly implicated in the occurrence of human ExPEC infections. For example, a recent longitudinal study of ~2,045 *E. coli* genomes (215 STs across all *E. coli* phylogroups) characterized by long read sequencing from patients with BSI identified frequent carriage of ColV plasmids [42], with another study identifying *E. coli* carrying ColV F plasmids as associated with 19.6% of patients with BSI [30]. This same study identified 23.9% of BSI patients carried *senB*-associated plasmids, with pandemic ExPEC STs including ST131-*H*30R1, ST131-*H*22 and ST1193 known to carry *senB*-associated plasmids [12,79].

### ColV-like and *senB*-associated F plasmids are present in instances of inter-source phylogenomic clusters

Our analyses identified a small number of plasmid clusters which were disproportionately common and associated with potential inter-source phylogenomic clusters (genomes differing by ≤10 cgMLST alleles). Primarily, reference plasmids from these plasmid clusters encoded (i) ColV-associated virulence genes, such as aerobactin and salmochelin siderophore operons – plasmid clusters AA176, AA175 and AA179 (with variable RSTs but predominantly F18:A-:B1), or (ii) *cjrABC-senB-*associated genes – plasmid clusters AA337 and AA171. Reference plasmids and putative plasmids residing within clusters AA337 and AA171 were structurally and genotypically similar to plasmid pUTI89 (F29:A-:B10) and pG150 (F1:A2:B20), both of which are strongly linked with extraintestinal virulence [38]. Consistent with our observations, Arredondo *et al*. [42] identified ColV- and *cjrABC-senB*-associated plasmid types in a long-read survey of 2,000 long-read *E. coli* isolates and showed their occurrence reflects structured associations between plasmid clusters and phylogenetic background.

The overrepresentation of putative plasmids from these plasmid clusters across *E. coli* from multiple hosts indicates their importance in shaping the ExPEC plasmidome and the potential for cross-species dissemination. ColV-associated plasmids have been repeatedly identified in APEC and are widespread within poultry systems [80,81] and contaminate retail poultry [9,76, 80]. These plasmids carry conserved virulence loci that promote colonization and systemic infection in poultry [80] as well as humans [82,83]. In contrast, the epidemiology of *cjrABC-senB-*associated plasmids is less well understood, aside from their known association with human ExPEC [30]. Research suggests that bacteriocins also play a major role in shaping the success of plasmid lineages as well as pandemic ExPEC lineages [38,42] – it is notable that both ColV- and *cjrABC-senB*-associated plasmids are widespread and both carry bacteriocins.

The dominance of these virulence-associated F plasmid lineages across the One Health interface has direct implications for surveillance. We also highlight that reference plasmid databases already contain examples of plasmids from such clusters which also carry genes conferring resistance to critically important antimicrobials, highlighting their dual role in disseminating both virulence and resistance determinants. Monitoring the prevalence and mobility of plasmids across One Health contexts will be critical for anticipating the emergence of CIA-resistant ExPEC lineages with zoonotic potential and designing interventions to mitigate their spread. Further research is required to shed light on the dynamics of inter-source plasmid mobilization. Pangenomic analysis by Matlock *et al*. on 1,458 fully assembled *Enterobacterales* genomes suggests that conserved plasmid backbones may move between diverse hosts and niches, in the process acquiring or losing accessory elements, and that these events may occur more frequently than previously thought [84].

### Study limitations and future directions

Due to the opportunistic nature of our sampling strategy, our study has several limitations: (i) human isolates, particularly those of clinical origin, are overrepresented within our collection, especially pandemic ExPEC lineages. Given that the top 20 ExPEC STs are responsible for more than 80% of the ExPEC disease burden [37], coupled with the association of plasmid lineages with particular ExPEC STs, this likely influences the predominant plasmid clusters we observed in our analysis; (ii) sample selection criteria were unavailable for the majority of isolates; and (iii) some states and territories of Australia as well as time periods and sources are underrepresented. It should also be noted that due to many of the genomes under analysis originating from public health labs and veterinary studies on food animals, a greater frequency of genomic clusters biases our collections phylogenetic distribution. A prospective genomic study of *E. coli* framed by a rigorous sampling regime that ensures wider representation of non-human isolates may prove insightful in documenting potential inter-source transmission events and the identification of rarer plasmid types with One Health significance.

The identification of unclustered plasmids, particularly among non-human-sourced strains, indicates a paucity of plasmids from such sources in this database (a shortcoming of databases more generally given the somewhat clinical-centric nature of microbial genomics). It also remains unclear whether plasmid reconstruction methods perform consistently for strains carrying plasmids that are poorly represented in available reference databases; resolving this will require dedicated benchmarking across diverse and under-represented plasmid lineages. A comprehensive understanding of plasmid epidemiology ultimately requires long-read sequencing, particularly given the frequency of plasmid rearrangements, which can confound inferences of genetic relatedness based on short-read data alone. However, we have shown that large, short-read datasets can still be informatively mined to infer ST- and lineage-specific plasmid associations [30]. This is valuable given the large volume of short-read sequence data in public databases, the majority of which is unlikely to ever be supplemented with complementary long-read sequence data but still can provide epidemiologically useful insights.

## Conclusion

Here, we investigated the presence of the two major ExPEC F plasmid lineages, one carrying ColV virulence genes and the other *cjrABC-senB* virulence cargo across a large national *E. coli* genomic dataset from widespread sources. Importantly, we demonstrate the existence of examples of such plasmid clusters carrying ARGs, including genes encoding resistance to antibiotics of critical importance to human health. While both F plasmid lineages are critical in ExPEC disease [30,42], our cluster analyses indicated that ColV cluster AA176 spans a broader One Health spectrum with a definitive association with food animal production, particularly poultry. F clusters that carry *cjrABC-senB* virulence cargo also span the One Health spectrum but, for reasons that are not well understood, are noticeably absent from food animal production reservoirs. Many novel putative plasmids were identified, particularly among non-human-sourced *E. coli*, highlighting the need for additional representation of the non-human *E. coli* plasmidome in global plasmid databases. Enhanced mining of short-read sequences using novel plasmid reconstruction and clustering algorithms, coupled with expanding genomic databases to include non-human *E. coli* plasmids, will be pivotal for delineating host-range patterns, understanding transmission dynamics and mitigating the spread of pandemic and emergent ExPEC across the One Health interface.

## Supplementary material

10.1099/mgen.0.001669 Uncited Supplementary Material 1.

## Abbreviations

ARG: antimicrobial resistance gene
BSI: bloodstream infection
cgMLST: core-genome multilocus sequence type
CIA: critically important antibiotic
ExPEC: extraintestinal pathogenic *Escherichia coli*
IQR: interquartile range
MDR: multiple drug resistant
MLST: multilocus sequence type
pMLST: plasmid multilocus sequence type
RST: F replicon sequence type
ST: sequence type
UTI: urinary tract infection
VAG: virulence-associated gene
