# Supplementary material for: The Escherichia coli F plasmidome: an Australian perspective
Source: Microb Genom. 2026 Apr 24;12(4):001669. doi: 10.1099/mgen.0.001669 (PMC13108677; doi:10.1099/mgen.0.001669)
Supplement: Uncited Supplementary Material 1. [file mgen-12-01669-s001.pdf]

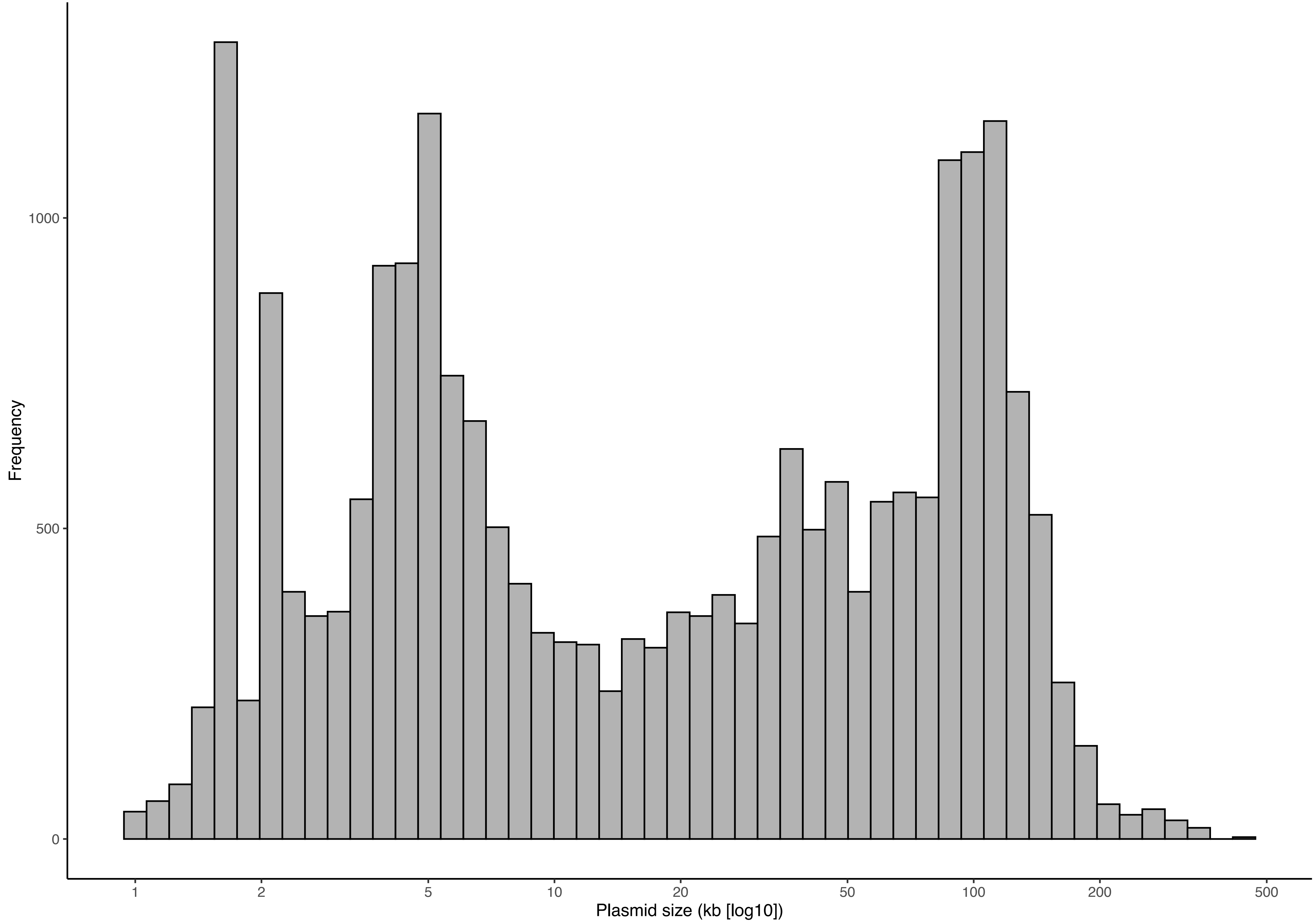

Frequency
1000
500
0
1
2
5
10
20
50
100
200
500
Plasmid size (kb [log10])

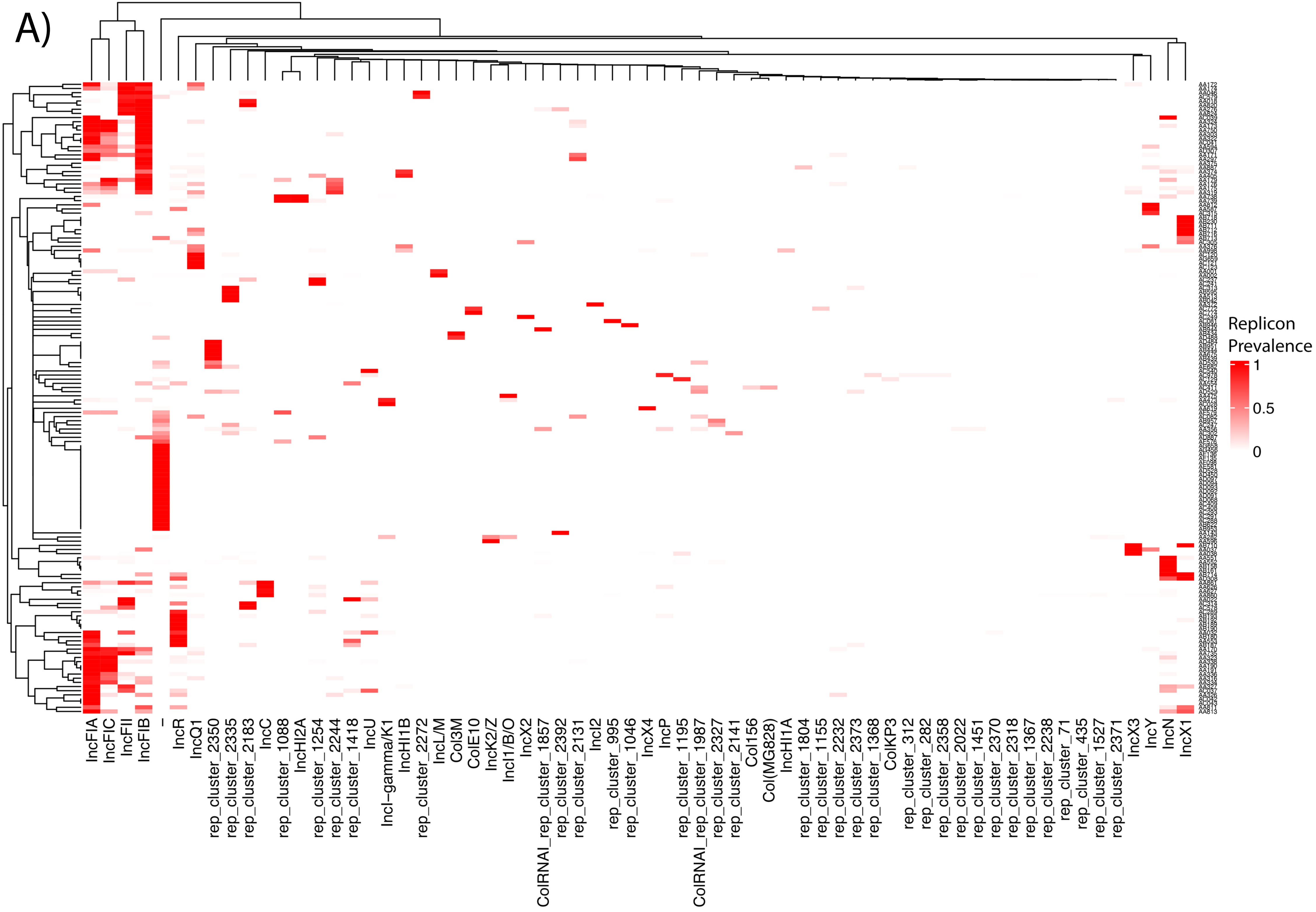
A)
Replicon
Prevalence
1
0.5
0
IncFIA
IncFIC
IncFII
IncFIB
–
IncR
IncQ1
rep_cluster_2350
rep_cluster_2335
rep_cluster_2183
IncC
rep_cluster_1088
IncHI2A
rep_cluster_1254
rep_cluster_2244
rep_cluster_1418
IncU
IncI–gamma/K1
IncHI1B
rep_cluster_2272
IncL/M
Col3M
ColE10
IncK2/Z
IncI1/B/O
IncX2
ColRNAI_rep_cluster_1857
rep_cluster_2392
rep_cluster_2131
IncI2
rep_cluster_995
rep_cluster_1046
IncX4
IncP
rep_cluster_1195
ColRNAI_rep_cluster_1987
rep_cluster_2327
rep_cluster_2141
Col156
Col(MG828)
IncHI1A
rep_cluster_1804
rep_cluster_1155
rep_cluster_2232
rep_cluster_2373
rep_cluster_1368
ColKP3
rep_cluster_312
rep_cluster_282
rep_cluster_2358
rep_cluster_2022
rep_cluster_1451
rep_cluster_2370
rep_cluster_2318
rep_cluster_1367
rep_cluster_2238
rep_cluster_71
rep_cluster_435
rep_cluster_1527
rep_cluster_2371
IncX3
IncY
IncN
IncX1

B)

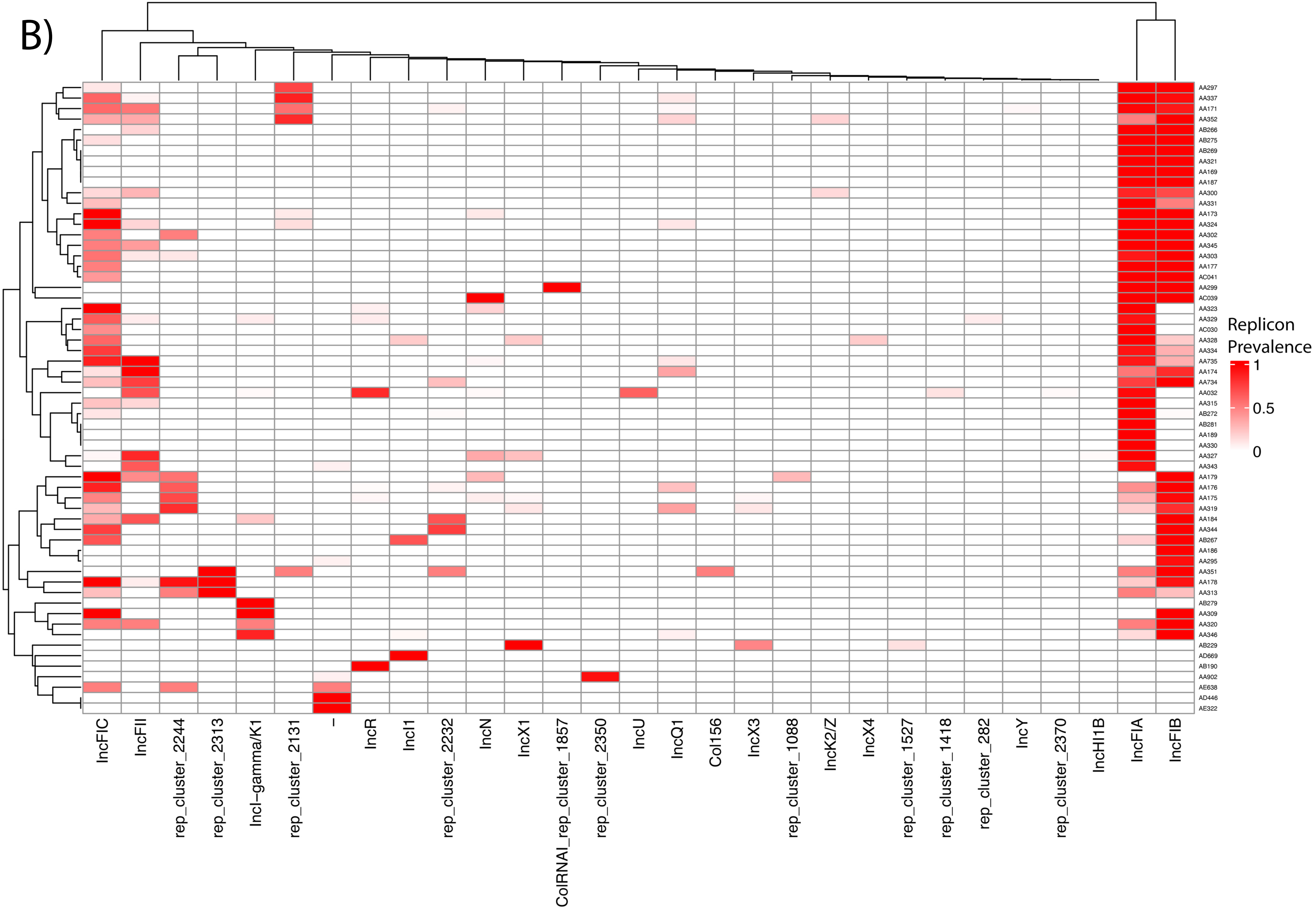

IncFIC
IncFII
rep_cluster_2244
rep_cluster_2313
IncI-gamma/K1
rep_cluster_2131
-
IncR
IncI1
rep_cluster_2232
IncN
IncX1
ColRNAI_rep_cluster_1857
rep_cluster_2350
IncU
IncQ1
Col156
IncX3
rep_cluster_1088
IncK2/Z
IncX4
rep_cluster_1527
rep_cluster_1418
rep_cluster_282
IncY
rep_cluster_2370
IncHI1B
IncFIA
IncFIB
Replicon Prevalence
1
0.5
0

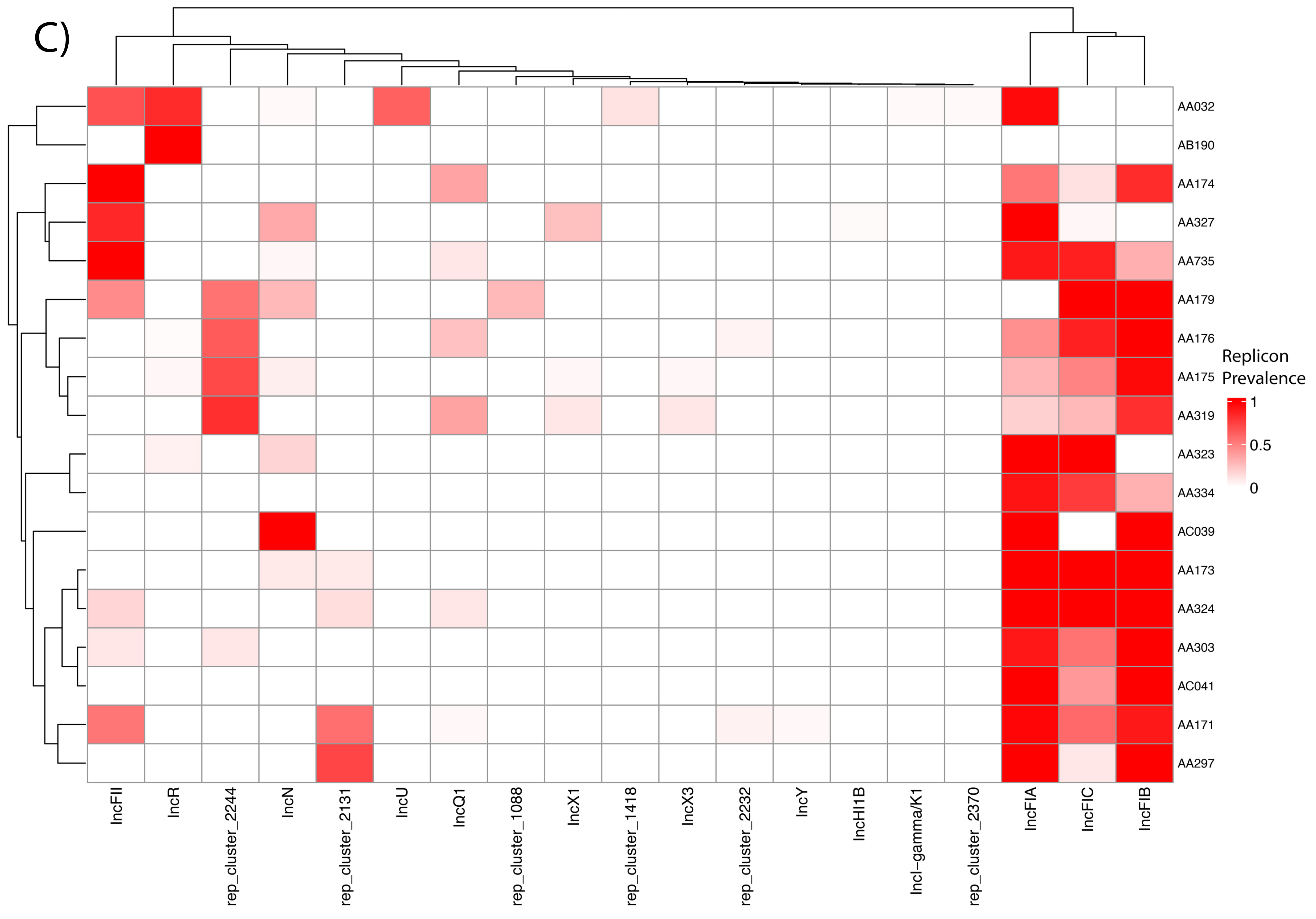
C)
AA032
AB190
AA174
AA327
AA735
AA179
AA176
AA175
AA319
AA323
AA334
AC039
AA173
AA324
AA303
AC041
AA171
AA297
IncFII
IncR
rep_cluster_2244
IncN
rep_cluster_2131
IncU
IncQ1
rep_cluster_1088
IncX1
rep_cluster_1418
IncX3
rep_cluster_2232
IncY
IncHI1B
IncI-gamma/K1
rep_cluster_2370
IncFIA
IncFIC
IncFIB
Replicon Prevalence
1
0.5
0

A)

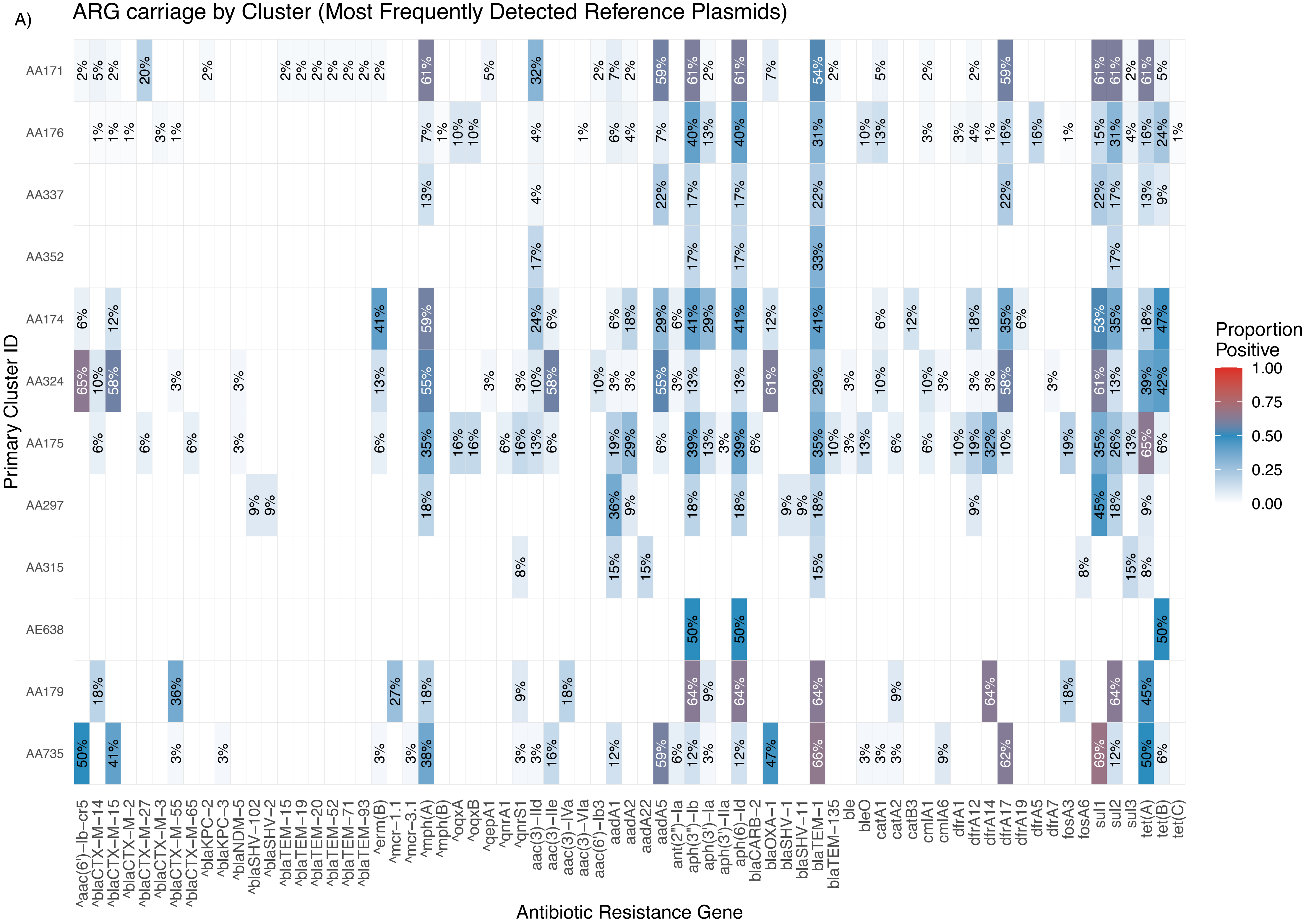
ARG carriage by Cluster (Most Frequently Detected Reference Plasmids)
Primary Cluster ID
Antibiotic Resistance Gene
Proportion Positive
1.00
0.75
0.50
0.25
0.00
AA171
AA176
AA337
AA352
AA174
AA324
AA175
AA297
AA315
AE638
AA179
AA735

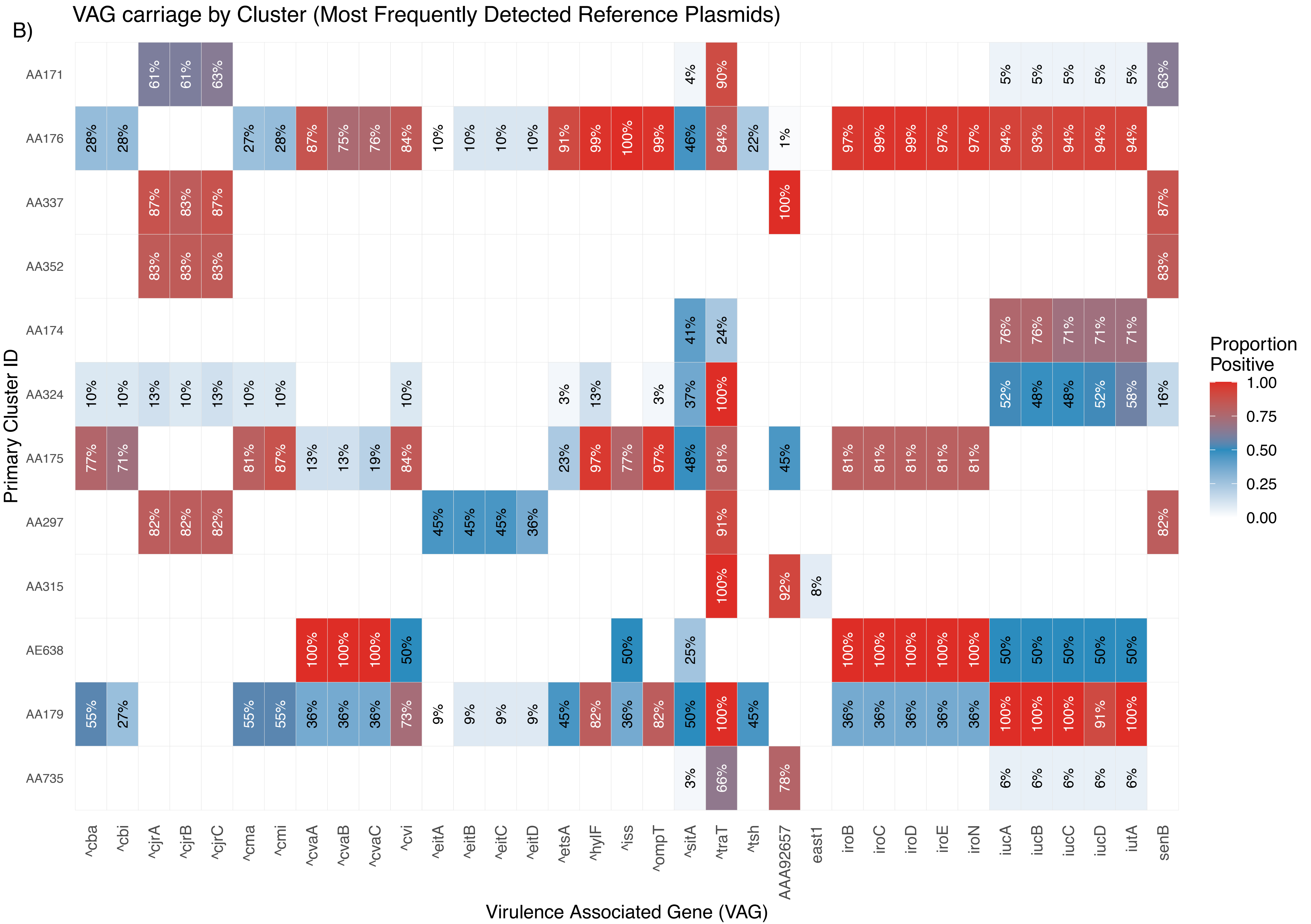
B)
VAG carriage by Cluster (Most Frequently Detected Reference Plasmids)
Primary Cluster ID
AA171
AA176
AA337
AA352
AA174
AA324
AA175
AA297
AA315
AE638
AA179
AA735
61% 61% 63% 4% 90% 5% 5% 5% 5% 5% 63%
28% 28% 27% 28% 87% 75% 76% 84% 10% 10% 10% 10% 91% 99% 100% 99% 46% 84% 22% 1% 97% 99% 99% 97% 97% 94% 93% 94% 94% 94%
87% 83% 87% 100% 87%
83% 83% 83% 83%
41% 24% 76% 76% 71% 71% 71%
10% 10% 13% 10% 13% 10% 10% 10% 3% 13% 3% 37% 100% 52% 48% 48% 52% 58% 16%
77% 71% 81% 87% 13% 13% 19% 84% 23% 97% 77% 97% 48% 81% 45% 81% 81% 81% 81% 81%
82% 82% 82% 45% 45% 45% 36% 91% 82%
100% 92% 8%
100% 100% 100% 50% 50% 25% 100% 100% 100% 100% 100% 50% 50% 50% 50% 50%
55% 27% 55% 55% 36% 36% 36% 73% 9% 9% 9% 9% 45% 82% 36% 82% 50% 100% 45% 36% 36% 36% 36% 36% 100% 100% 100% 91% 100%
3% 66% 78% 6% 6% 6% 6% 6%
^cba ^cbi ^cjrA ^cjrB ^cjrC ^cma ^cmi ^cvaA ^cvaB ^cvaC ^cvi ^eitA ^eitB ^eitC ^eitD ^etsA ^hylF ^iss ^ompT ^sitA ^traT ^tsh AAA92657 east1 iroB iroC iroD iroE iroN iucA iucB iucC iucD iutA senB
Virulence Associated Gene (VAG)
Proportion Positive
1.00
0.75
0.50
0.25
0.00

Supplementary Figure 1 - Distribution of putative plasmid sizes in the study collection. Histogram showing the size distribution of putative plasmids identified across the dataset. Plasmid size is shown in kilobases (kb) on a log10 scale to accommodate the wide dynamic range observed. The distribution is multimodal, reflecting the presence of both small plasmids and larger plasmid types, including F-type plasmids, within the collection.

Supplementary Figure 2 – Replicon carriage among AMR-associated primary plasmid clusters from the MOBSuite database. A) Only primary plasmid clusters for which ≥60% of representatives carried one or more resistance gene are shown. B) Only primary plasmid clusters for which ≥60% of representatives carried one or more virulence associated gene are shown. C) Only primary plasmid clusters for which ≥60% of representatives carried both one or more resistance gene and one or more virulence gene are shown. The proportion of representatives of a given cluster carrying a give replicon is indicated by colour.

Supplementary Figure 3 – A) Aggregated carriage of antimicrobial resistance genes (ARGs) among reference plasmid sequences (by cluster) from the reference plasmid database. ^ - Genes associated with resistance to critically important antimicrobials. B) Aggregated carriage of virulence associated genes (VAGs) among reference plasmid sequences (by cluster) from the reference plasmid database. ^ - Genes were sourced from a custom nucleotide database (see methods); genes not marked as such were sourced from VFDB.

Supplementary Table 1 – Biosample accession numbers, metadata, genotypic data and primary plasmid cluster carriage for genomes under analysis (File too large for inclusion – see https://github.com/maxlcummins/APG-OHEC-Retro-M2).

Supplementary Table 2 – Accession numbers, primary plasmid cluster associations and genotypic data for MOB-suite reference database plasmids under analysis (File too large for inclusion – see https://github.com/maxlcummins/APG-OHEC-Retro-M2).
